# An *in silico* study to find potential effective circRNAs in the progression of Huntington’s disease

**DOI:** 10.22038/IJBMS.2023.67791.14839

**Published:** 2023

**Authors:** Anahita Moradi, Farbod Shahabinezhad, Ali Dehshahri

**Affiliations:** 1 Pharmaceutical Sciences Research Center, Shiraz University of Medical Sciences, Shiraz, Iran; 2 Department of Pharmaceutical Biotechnology, School of Pharmacy, Shiraz University of Medical Sciences, Shiraz, Iran

**Keywords:** Bioinformatics, Circular RNA (circRNA), Huntington’s disease Micro, RNA (miRNA), Neurodegenerative-disorders

## Abstract

**Objective(s):**

Huntington’s disease (HD) is identified as a progressive genetic disorder caused by a mutation in the Huntington gene. Although the pathogenesis of this disease has not been fully understood, investigations have demonstrated the role of various genes and non-coding RNAs in the disease progression. In this study, we aimed to discover the potential promising circRNAs which can bind to miRNAs of HD.

**Materials and Methods:**

We used several bioinformatics tools such as ENCORI, Cytoscape, circBase, Knime, and Enrichr to collect possible circRNAs and then evaluate their connections with target miRNAs to reach this goal. We also found the probable relationship between parental genes of these circRNAs and the disease progress.

**Results:**

According to the data collected, more than 370 thousand circRNA-miRNA interactions were found for 57 target miRNAs. Several of circRNAs were spliced out of parental genes involved in the etiology of HD. Some of them need to be further investigated to elucidate their role in this neurodegenerative disease.

**Conclusion:**

This *in silico* investigation highlights the potential role of circRNAs in the progression of HD and opens up new horizons for drug discovery as well as diagnostic approaches for the disease.

## Introduction

Huntington’s disease (HD) is one of the most progressive neurodegenerative diseases. While a healthy human possesses less than 30 CAG trinucleotide repeats in the gene HUGO: 4p16.3, the patients carry 36-121 repeats per gene (1, 2). This simple genetic defect results in various neurodegeneration symptoms such as motor and cognitive malfunctioning, and various psychiatric disorders (3). Most Huntington’s patients develop their first symptoms when they reach their 40’s, and after that, the symptoms develop and progress into being more severe and noticeable. However, in some patients, the symptoms develop faster, while in some cases the onset happens at higher ages (4). It is hypothesized that some defects caused by aging mechanisms trigger the onset of HD progression (5). 

Recently, great attention has been directed to microRNAs as biomarkers. Several reports are indicating the biomarker roles of miRNAs in various diseases such as malignancies (6), diabetes (7), cardiovascular (7), autoimmune (8), or neurodegenerative diseases (9). Fundamentally, miRNAs are byproducts of different gene expressions and are regularly produced in the cells. Since the gene expression profiles of the cells may change in a disease, the expression of these miRNAs can be altered and these changes could be considered as key biomarkers of the pathological conditions (6, 10).

 There are several reports on the Differentially Expressed miRNAs (DEMs) in HD patients. While the objective of most studies is focused on the DEMs between healthy and symptomatic Huntington’s patients, there are two reports on miRNA expressions both in prodromal and symptomatic individuals, as well as the healthy ones (11, 12). Although there is extensive data on healthy *vs* symptomatic Huntington’s patients, this information cannot be directly used to discover the hidden mechanisms associated with the progression of the disease. In order to figure out the relationship of circRNAs with the progression of the disease, each patient carrying the malfunctioning genetic codes should be analyzed prior to and after the onset of their symptoms in a very long and time-consuming study. Following data gathering and analysis, we can rely on existing research comparing DEMs in presymptomatic and symptomatic Huntington patients. 

Although miRNAs could be considered biomarkers, their application as drug candidates is not promising due to their unstable nature (13). On the other hand, their functions can be regulated by more stable, smaller non-coding circRNAs. There are extensive studies investigating the targeting of various functions of circRNAs to modify their regulatory functions (14). 

CircRNAs are the products of the back-splicing of exons in mRNA precursors. The 5’ and 3’ ends of mRNA precursors are covalently linked together to form a circular non-coding nucleic acid material (14). Despite the non-coding nature of such material, this structure can play crucial roles in mammalian cell functions (15). One of the interesting functions which are attributed to circRNAs is their binding to miRNAs and gene expression regulation. Their main mechanism of action is called “miRNA sponging” (16). A single circRNA can contain several binding sites for one or multiple miRNAs competing for those binding sites. Hence, they can block the gene expression inhibitory roles of miRNAs resulting in the regulation of their functions (16). 

Due to their unique characteristics, the role of circRNAs has been extensively studied in a wide variety of diseases, including neurodegenerative disorders (17). For instance, Zhang *et al*. (18) have investigated the regulatory functions of circRNAs in Alzheimer’s disease, where they have explained some of the main mechanisms of action of circRNAs such as miRNA sponging to reduce neuroinflammation and oxidative stress, and regulation of amyloid-beta (Aβ) generation in the brain tissue. Similarly, this role has been studied in Parkinson’s disease and its possible neuroprotective effects (19). Various investigations have illustrated the importance of *in* *silico* analysis before conducting in-lab experiments (20). Today, these studies are not restricted to DNA sequencing but also extended to the various areas of genomics (21), proteomics (22), and RNA biology as well (23).

In this study, we analyzed the reported miRNAs related to HD and mapped their interactions with their interacting circRNAs. The results demonstrated multiple circRNAs with high interaction with the extracted miRNAs. These results could be used as an *in silico* basis for further *in vitro* and *in vivo* investigations to find circRNAs in HD. 

## Materials and Methods

***Data gathering***

The results of two previous reports by Hoss *et al*. and Reed *et al.* (11, 12) concerning the miRNAs of HD patients compared with healthy individuals were extracted. Hoss *et al.* (24) profiled the miRNA expression of 26 symptomatic HD patients and 36 healthy individuals by the GEO Series accession number of GEO: GSE64977. Reed *et al.* (12) profiled differentially expressed miRNAs in 14 HD-diagnosed patients compared with 14 healthy individuals. Additionally, they used samples of 14 diagnosed, 10 low-risk, 8 medium-risk, 10 high-risk participants, and 14 healthy controls to measure the expression level of miRNAs. In this study, both prodromal and postmortem HD patients’ sets of miRNAs were analyzed and compared with healthy individuals. We also chose differentially expressed genes in HD reported in another study for further comparisons (25). 

***Establishing miRNA-circRNA interactions***

The gathered data were manually put into the ENCORI web server (26) to interpret known and valid interacting circRNAs. ENCORI is an open-source platform for studying the miRNA-ncRNA, miRNA-mRNA, ncRNA-RNA, RNA-RNA, RBP-ncRNA, and RBP-mRNA interactions from CLIP-seq, degradome-seq, and RNA-RNA interactome data. We used circBase (27) IDs as the main identification method of circRNAs to interpret ENCORI results. 

The result of ENCORI analysis has been shown as a spreadsheet for each entered miRNA ID with its interacting circRNAs (circBase IDs) and relevant genes (see Supplementary Table 3). In order to achieve automation and reduce manual data input errors, ENCORI web API (curl) was used in the HTTP Retriever node of the Knime application, an open-source program used to perform batch calculations using its extensive pool of extensions and nodes. In other words, a unique URL was built for each miRNA ID and downloaded by the Http Retriever node to be organized and put together with the remaining data in Knime. The URL comprised one unique miRNA ID, such as “hsa-miR-17-5p”, and relevant information such as gene type and limiting filters. In the end, the built URL was similar to this example for the above-mentioned miRNA ID: 

http://starbase.sysu.edu.cn/api/miRNATarget/?assembly=hg19&geneType=circRNA&miRNA=hsa-miR-17-5p&clipExpNum=1&degraExpNum=0&pancancerNum=10&programNum=5&program=None&target=all&cellType=all.

After the data was reorganized with Microsoft Excel 2013, the circRNAs IDs were sorted based on the frequency of interaction repeats with miRNAs. A circRNA might have been reported to interact with the same miRNA by several different target sequences. In this case, each target sequence was counted as a separate interaction.

A table containing column names of miRNA ID, Gene ID, and circRNAs ID of all entered miRNAs was generated with sorted circRNA counts based on the frequency of repeats. 

***Drawing networks of interactions***

The final table was generated from the data obtained in the previous table containing only the circRNAs interacting with more than 100 miRNAs according to the results gathered from the ENCORI database (Supplementary Table 1). Each row of the table represents the interaction of a binding site of a single circRNA to a miRNA. In this table, circRNAs are sorted based on the prevalence of their interactions with miRNAs. 

The data of this table was analyzed with the Cytoscape program (28) to draw a network map of interactions between miRNAs and their interacting circRNAs. Each node has been colored based on the degree of interactions after network analysis by the Cytoscape program. In order to minimize the number of used nodes in the drawn networks, the interactions of circRNAs with more than 100 repeats were exclusively imported into the network of circRNA-miRNA. Additionally, differentially expressed genes of HD patients were obtained and compared with the table containing circRNAs data. circRNAs derived from those DEGs were separately mapped with their interacting miRNAs in the miRNA-parental gene of the circRNAs network. To have a clear view of the interacting circRNAs of those parental genes and target miRNAs, we examined down-regulated and up-regulated genes in two maps independently. 

***Gene Ontology and KEGG pathway enrichments analysis***

Parental genes of collected circRNAs were selected to analyze via Enrichr (29) (http://amp.pharm.mssm.edu/Enrichr/), a convenient instrument to obtain functional annotation and biological information of genes. Retrieved GO terms (30) and KEGG (31) pathways with a *P*-value<0.05 were considered to be significantly enriched.

## Results

***miRNA selection and prediction of the potential circRNA binding to miRNAs in HD***

We selected 81 miRNAs from Hoss *et al*. study and 35 miRNAs from Reed *et al*. investigation (Supplementary Table 2). In order to construct miRNA-circRNA networks, all target miRNAs were inserted into the starBase v2.0 database, and more than 370 thousand interactions were found for 57 target miRNAs. Supplementary Table 1 demonstrates the top forty circRNAs interacting with target miRNAs. Evidently, circBase: hsa_circ_8167, hsa_circ_0005283, hsa_circ_0003603, circRNAs derived from the HUGO: *CLIP2* gene, provided the most significant number of binding sites for target miRNAs with 558, 556, and 555 binding regions for 56 miRNAs, respectively. In detail, hsa-miR-150-5p, hsa-miR-106a-5p, hsa-miR-302a-3p, hsa-miR-761, and hsa-miR-10b-5p were the top five miRNAs that these three circRNAs made the most bonds with. Likewise, these miRNAs had the most interactions with hsa_circ_0009129 splicing out of the *GTF2I* gene. Moreover, this pattern was almost similar for the other circRNAs of this gene, hsa_circ_0003317, and hsa_circ_0006821.

Nevertheless, hsa-miR-670-3p, hsa-miR-302a-3p, hsa-miR-106a-5p, and hsa-miR-224-5p had the highest number of connections with four circRNAs derived out of the *NBPF10* gene. 

***miRNA-circRNA comprehensive regulatory networks***

**Figure 1:**
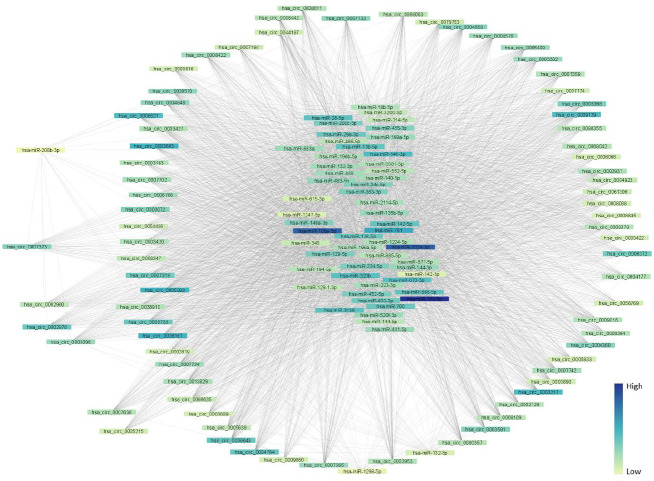
miRNA-centered regulatory network of circRNA-miRNA interaction. The color range shows the level of interactions between potential circRNAs and target miRNAs

More than 65 thousand unique circRNAs were able to act as sponges for target miRNAs of HD. To demonstrate the circRNA-miRNA interactions, a miRNA-centered regulatory network of 77 circRNAs constructing more than a hundred connections with target miRNAs was assembled (Figure 1). The results showed that three up-regulated miRNAs, miRBase: hsa-miR-150-5p, hsa-miR-302a-3p, and has-miR-106a-5p, had the highest number of edges with selected circRNAs. According to the results, miRBase: miR-150-5p had 46 binding sites on circBase: circ_0008167, and both miRBase: miR-106a-5p and miR-302a-3p were able to bind 36 regions on this circRNA (Supplementary Table 3).

***Identification of circRNAs spliced out of differentially expressed genes binding to target miRNAs in HD patients compared with the healthy controls***

To find out the possible regulatory effects of circRNAs derived from differentially expressed genes (DEGs) in HD, 167 DEGs (99 up-regulated and 68 down-regulated genes) were obtained from GEO: GSE51799 (25). According to data collected from ENCORI, 650 unique circRNAs spliced out of 50 down-regulated DEGs, and 261 distinctive circRNAs were constructed from 46 up-regulated DEGs (Supplementary Table 3). Among the circRNAs of those down-regulated DEGs, 19 circRNAs spliced out of the *BAZ1B* gene, having 367 circRNA-miRNA linkages, and also 266 of those connections belong to hsa_circ_0008576, which had the highest number of interactions among all the circRNAs related to down-regulated DEGs and attached to 51 out of 57 target miRNAs. This circRNA had only 26 interactions with hsa-miR-150-5p and 15 with hsa-miR-761 and hsa-miR-106a-5p. hsa_circ_0009016 and hsa_circ_0023967, the circRNAs spliced out of the *PRSS23* gene, had 202 and 12 connections with 49 and 10 target miRNAs. The highest number of interactions for the former circRNA was 12 with hsa-miR-302a-3p.

 Eighty-one circRNAs originated from the* MACF1* gene creating the highest number of interactions among down-regulated DEGs and had linkages with 32 target miRNAs. These 81 circRNAs had 1235 connections with target miRNAs, which was significant. circRNAs acquired from this gene had the most linkages with hsa-miR-670-3p and hsa-miR-452-5p.

 The *MDN1 *gene was another with many circRNAs, 70 distinct circRNAs having 729 overall interactions with 23 target miRNAs. The data relating to these circRNAs has shown that 37 of them built 84 connections only with hsa-miR-431-5p, 48 of them made 58 connections with hsa-miR-670-3p, and 37 constructed 58 networks with hsa-miR-129-5p. 

Moreover, forty-three unique circRNAs of the *UBAP2L* gene had 245 attachments with 16 unique miRNAs, and among the target miRNAs, hsa-miR-877-5p and hsa-miR-760 were the ones with the most connections with those circRNAs.

The next group of circRNAs is acquired from the *GPI* gene, which was 31 circRNAs with 170 connections. *CKAP5 *and *MCM3* genes built 34 and 29 individual circRNAs and were predicted to build 115 and 159 circRNA-miRNA networks. This number was 58 for the *MTOR* gene’s circRNAs and 322 for connections with 14 distinct miRNAs. *PTPN4* and *SFMBT2* genes were parental genes for seventeen and twenty-five circRNAs constructing 64 and 46 interactions with only 10 and 6 miRNAs. *HEATR5B* gene was the following parental gene on the list, being the origin of 55 different circRNAs creating 290 connections with 12 particular miRNAs. Nineteen and fourteen distinct circRNAs originated from *FANCI* and *CHD6* genes and made 79 and 45 linking sites. Nine circRNAs were made from *MAGED1 *and *HEATR2* genes and ten from *SDHA* genes which built 67, 28, and 32 connections. The rest of the parental down-regulated genes had considerably fewer connections, such as *STARD9, CEP72, AGK, LAMTOR1, SLC25A33, ABCD4, ZNHIT6, AHNAK, HIVEP3, ARL4C, ZNF562, FYN, TMEM109, GNPTAB, GNPNAT1, KCTD10, NMT2, ZNF320, PAN2, TTC38, TFDP2, GPR56, BOLA1, SUN2, PTCH1, RIOK1, SART3, ACAD9, RASA3, OTUD7B, ARAP2, TAF15,* and C2orf42 genes.

**Figure 2:**
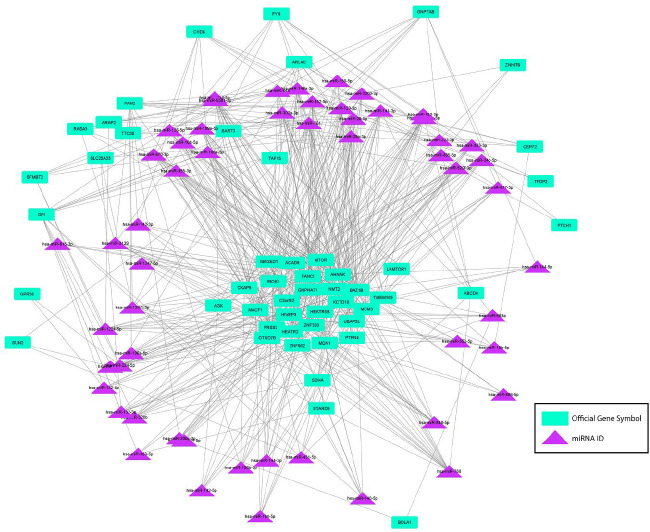
A regulatory network of 50 down-regulated DEGs in HD patients as parental genes of detected circRNAs binding to target miRNAs. Purple triangles represent miRNAs and green rectangles represent parental genes

**Figure 3:**
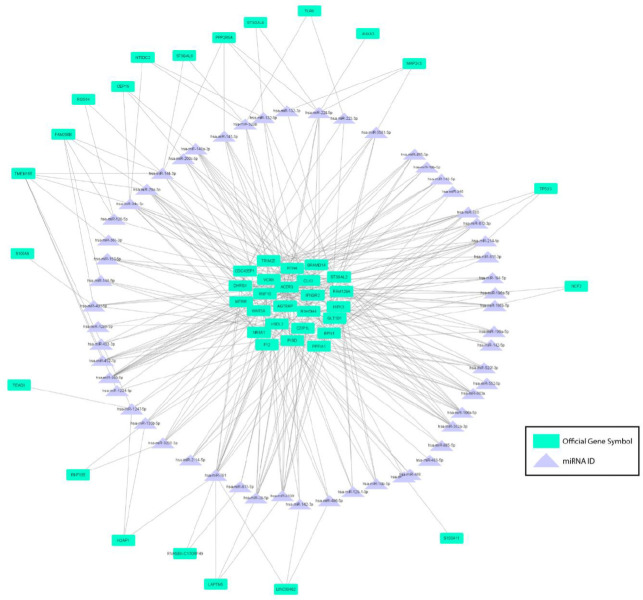
A regulatory network of 46 up-regulated DEGs in HD patients as parental genes of detected circRNAs binding to target miRNAs. Purple triangles represent miRNAs and green rectangles represent parental genes

The number of interactions in circRNAs spliced out of the up-regulated DEGs was considerably lower than the number for down-regulated genes. As the data showed, circRNAs derived from *VCAN*, *RNF10, TRIM25, PPFIA1, RPN1, HIPK3, WNT5A,* and *RTN4* genes constructed 129, 119, 106, 95, 94, 88, 74, and 53 circRNA-miRNA networks, respectively. Accordingly, the nine unique circRNAs created from the *VCAN* gene could bind to 17 target miRNAs in which hsa-miR-106a-5p had the most connections. *RNF10 *gene was the parental gene of 26 circRNAs binding to 15 target miRNAs; hsa-miR-148a-3p and hsa-miR-760 had the highest interactions with these circRNAs. The data has shown that circRNAs related to the *RNF10* gene had the most interactions with hsa-miR-148a-3p and hsa-miR-760. However, hsa-miR-28-5p and hsa-miR-3139 were the two miRNAs interacting the most with *TRIM25* and *PPFIA1 *genes’ derived circRNAs. *RPN1* gene’s related circRNAs are connected to hsa-miR-452-5p and hsa-miR-148a-3p more than other target miRNAs. circRNAs of *HIPK3* genes had the most connections with hsa-miR-148a-3p and others like hsa-miR-10b-5p, hsa-miR-132-3p, and hsa-miR-552-5p. While these eight genes’ circRNAs interacted with nine to nineteen unique miRNAs, circRNAs of some genes like *LAPTM5 *made connections only to three miRNAs, hsa-miR-28-5p, hsa-miR-3139, and hsa-miR-761. The rest of the circRNAs of up-regulated parental genes showed fewer circRNA-miRNA bonds than the mentioned genes. Figures 2 and 3 illustrate the up- and down-regulated DEGs as parental genes of these circRNAs connected with objective miRNAs. 

***Functional enrichment analysis of parental genes***

**Figure 4:**
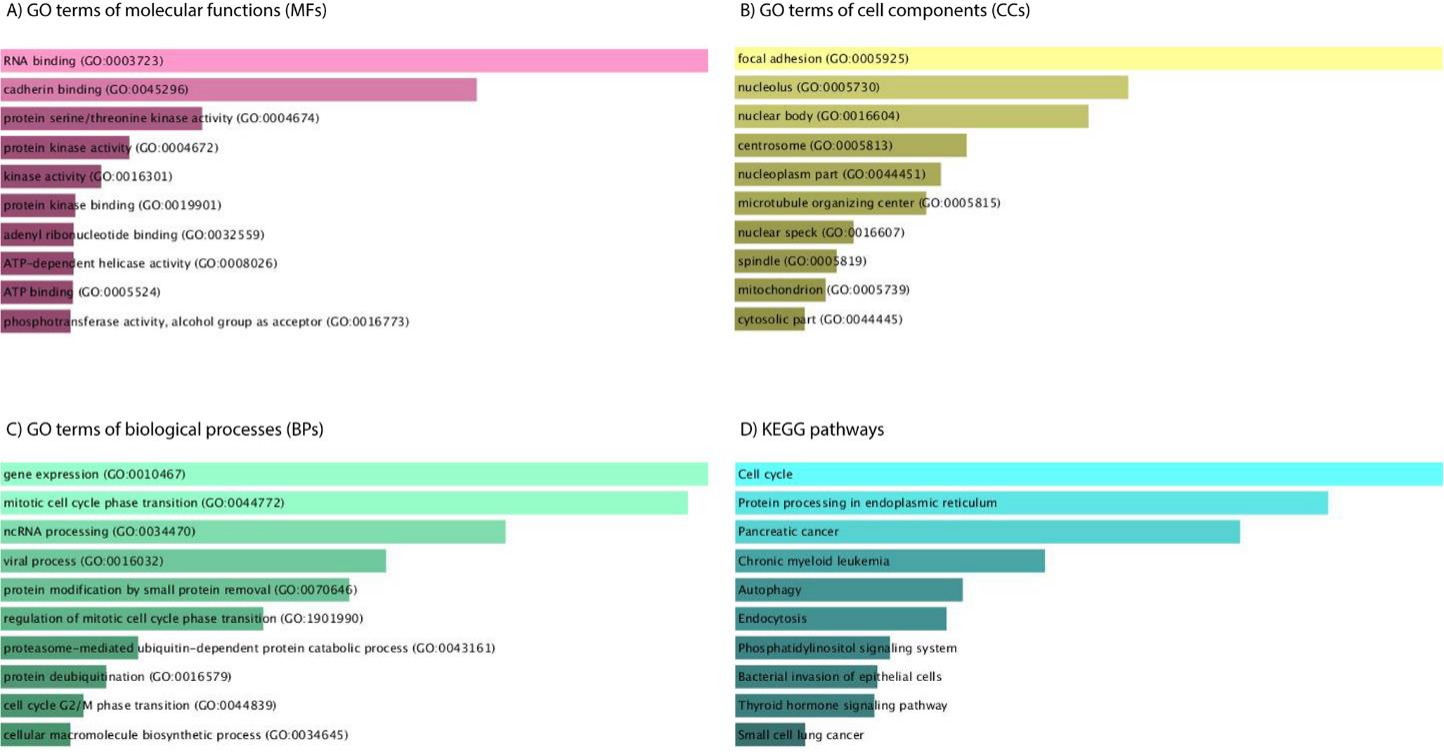
GO and KEGG pathway analysis of parental genes. GO analysis identified (A) molecular functions, (B) cell components, and (C) biological processes. (D) Relevant pathways were established for parental genes

To better understand the biological functions of parental genes, we performed Gene Ontology (GO) and Kyoto Encyclopedia of Genes and Genomes (KEGG) pathway enrichment analyses. The GO analysis results revealed the ten most relevant biological processes, molecular functions, and cell components of parental genes. It is observed that gene expression, mitotic cell cycle phase transition, and non-coding RNA processing were the top three considerably enriched biological processes (Figure 4C). In terms of molecular functions, the top three functions were RNA binding, cadherin binding, and protein serine/threonine kinase activity (Figure 4A). Focal adhesion, nucleolus, and nuclear body were the top three cellular components for parental genes (Figure 4B). Furthermore, KEGG pathway analysis indicated that cell cycle, protein processing in the endoplasmic reticulum, and pancreatic cancer pathways were the most significant ones among parental genes. 

## Discussion

HD is known as an inherited neurodegenerative disease caused by anomalous expansion of CAG repeats in exon 1 of *the Huntington* gene leading to excessive aggregation of mutant Huntington protein. These abnormal aggregates result in the dysregulation of mitochondrial and proteasome pathways, transcriptional regulation, and protein-protein interactions, and consequently give rise to neuronal cell death. In this regard, several investigations have been done to elucidate the precise mechanism of disease progression. Among those studies, a significant number of them aimed to clarify the role of ncRNAs in the etiology of HD, specifically finding miRNA-mRNA regulatory networks (32). However, there have been no attempts to determine the role of circRNAs in this genetic disorder so far. 

In this study, we selected all 81 differentially expressed miRNAs of next-generation miRNA sequence analysis of brain samples obtained from 26 HD patients and 36 healthy controls (11). Additionally, 35 differentially expressed miRNAs that have not been reported before in any studies were chosen from Reed *et al*. investigation. The analysis was conducted on 14 diagnosed HD patients and 14 healthy controls, and twenty-five miRNAs were reported as DEmiRNAs. We also chose this study because they found relevant miRNAs associated with ordinal categories of control, prodromal, and diagnosed HD. In this regard, they reported sixteen miRNAs (including the six differentially expressed miRNAs) whose expression is associated with various stages of HD. In other words, miRNAs with the lowest nominal *P*-values were selected (12). We studied all the miRNAs using the ENCORI database and obtained circRNA-miRNA interactions for 57 miRNAs in the next step.

The study conducted by Tan and colleagues on HD patients demonstrated that the transcription factor HUGO: *GTF2A1* appears to be dysregulated in competing endogenous RNA network interaction and is associated with transcriptional activation. Moreover, HUGO: *SFRP1* and *WNT3* genes probably play a role in the canonical Wnt/β-catenin signaling pathway, an essential constituent in the progress of neurodegenerative diseases. Another impaired pathway in such diseases is the calcium signaling pathway, and three genes, HUGO: *GNAS,* *CXCR4*, and *ITPKB *which seem to be dysregulated. Other dysregulated genes such as HUGO: *PTGS2*, *TRAF1*, and *MAP3K8 *may participate in the inflammatory responses, commonly in neurodegenerative disorders including HD (33). In our study, we identified circRNAs that derived from all these genes as well.

According to the data collected, differentially expressed genes may play a remarkable role in regulating target miRNAs of HD. There is supporting evidence that these genes are drastically associated with the motor score of HD patients (25). We observed that circRNAs spliced out of the down-regulated genes provided 4796 links in the miRNA-circRNA network. Indeed, 1236 connections were detected for up-regulated gene-derived circRNAs. Among them, circHUGO: circBAZ1B and circPRSS23 were highly connected to the target miRNAs.

Among potential circRNAs, we also found parental genes such as HUGO: *HIP1, SP1,* *PCNA, CHEK1, CCNA2, SIRT1,* and *REST* genes the roles of which in HD progression have already been confirmed. It has been shown that Huntingtin Interaction Protein 1 (UNIPROT: HIP1) cannot bind strongly to mutant Huntington protein leading to cell apoptosis (34). As for HUGO: *SP1*, it has been demonstrated that transcription factor SP1 promotes *Huntington* gene transcription resulting in abnormalities in the early stages of HD (35). An investigation has shown that HUGO: *CCNA2*, *PCNA*, AND *CHEK1 *genes are dysregulated by miRNAs resulting in cell cycle malfunction and apoptosis leading to neurotoxicity. A previous study showed that Sirtuin 1 (UNIPROT: SIRT1) is also involved in mitochondrial biogenesis and UNIPROT: brain-derived neurotropic factor (UNIPROT: BDNF) gene expression. Depletion of UNIPROT: BDNF is related to repressor element 1-silencing transcription factor (UNIPROT: REST), which appears to be involved in multiple mechanisms of HD pathogenesis (32).

HUGO: HOX genes-related circRNAs were also investigated in our study. A previous investigation showed that these genes may have indirect neuroprotective effects in HD (24).

A gene expression profiling of fibroblast cells revealed that HUGO: *PLCB4, UBE2D3A, APC, *and* ROCK1* genes are up-regulated in HD patients (36). In this regard, we observed that circRNAs derived from HUGO: *APC*, *ROCK1*, and *PLCB4* genes interact with target miRNAs. According to our results, circHUGO: circPDE10A made thirty connections with target miRNAs. Phosphodiesterase 10A is known as a promising biomarker of HD pathology (37). We also found several circRNAs spliced out of the HUGO: *E2F2 *gene associated with the age of onset in HD patients (38).

CircRNA data revealed that circRNAs derived from HUGO: *CLIP2* and *GTF2I* genes had the highest binding capacity for most miRNAs. Although there was no evidence to show the role of these two genes in HD, there are some studies indicating the contribution of these genes in the typical cognitive profile of a rare neurodevelopmental disorder (39). It is also shown that the HUGO: *CLIP2* gene plays a role in brain tumors (40). Neuroblastoma breakpoint family member 10 (UNIPROT: NBPF10) gene was another one with no contribution to HD, but four circHUGO: circNBPF10s developed a considerable number of connections with target miRNAs of HD. The parental gene of circHUGO: circRANBP2, another potential circRNA with a significant number of links, had no biological relevance to HD pathogenesis but was differentially expressed in glioblastoma (41).

## Conclusion

We showed that RNA binding was the leading molecular function of total parental genes. Most of them were involved in gene expression procedures which were the dominant biological process, so they might play a prominent role in regulating miRNAs in HD. To conclude, this study aimed to find potential circRNAs binding to key miRNAs of HD. We studied a substantial number of circRNAs spliced out of genes that were either involved in HD progression or not. These findings prove that we must consider the undeniable role of these non-coding RNAs in HD pathogenesis. Further *in vitro* and *in vivo* investigations are in demand to gain a deeper insight into the exact mechanism of action of these circRNAs in HD. 

## Authors’ Contributions

A M and F Sh designed the analysis and drafted the manuscript. A D supervised the project and edited the final manuscript.

## Funding

 This research did not receive any specific grant from funding agencies in the public, commercial, or not-for-profit sectors.

## Compliance with ethical standards

The article contains no research in which animals were used.

## Data Availability

The data used to support the findings of this study are available from the corresponding author upon request.

## Conflicts of Interest

The authors declare that they have no competing interests.
